# Multiplexed manipulation of orbital angular momentum and wavelength in metasurfaces based on arbitrary complex-amplitude control

**DOI:** 10.1038/s41377-024-01420-6

**Published:** 2024-04-28

**Authors:** Guoli He, Yaqin Zheng, Changda Zhou, Siyang Li, Zhonghong Shi, Yanhui Deng, Zhang-Kai Zhou

**Affiliations:** State Key Laboratory of Optoelectronic Materials and Technologies, School of Physics, Sun Yat-sen University, Guangzhou, 510275 China

**Keywords:** Nanophotonics and plasmonics, Metamaterials

## Abstract

Due to its unbounded and orthogonal modes, the orbital angular momentum (OAM) is regarded as a key optical degree of freedom (DoF) for future information processing with ultra-high capacity and speed. Although the manipulation of OAM based on metasurfaces has brought about great achievements in various fields, such manipulation currently remains at single-DoF level, which means the multiplexed manipulation of OAM with other optical DoFs is still lacking, greatly hampering the application of OAM beams and advancement of metasurfaces. In order to overcome this challenge, we propose the idea of multiplexed coherent pixel (MCP) for metasurfaces. This approach enables the manipulation of arbitrary complex-amplitude under incident lights of both plane and OAM waves, on the basis of which we have realized the multiplexed DoF control of OAM and wavelength. As a result, the MCP method expands the types of incident lights which can be simultaneously responded by metasurfaces, enriches the information processing capability of metasurfaces, and creates applications of information encryption and OAM demultiplexer. Our findings not only provide means for the design of high-security and high-capacity metasurfaces, but also raise the control and application level of OAM, offering great potential for multifunctional nanophotonic devices in the future.

## Introduction

Because of their on-demand control of the optical degree of freedom (DoFs) (such as wavelength, propagation direction, polarization, amplitude, phase, etc.) at the deep-subwavelength scale, metasurfaces have made a great contribution to the field of nanophotonics, integrated optics, quantum science^1–9^, etc. For example, since the metasurface has the capacity to precisely control the phase of propagating light, a field of metalens which functions as lenses in the microscale has emerged. The metalens possesses the advantages of easy fabrication and seamless integration into wearable devices or optoelectronic chips, greatly promoting the developments of fundamental research as well as industrial production fields, including virtual reality glasses, cell phone cameras, and portable spectrometers^10–13^. Furthermore, even the multiplexed control of two or more DoFs has been realized in a single metasurface, which provides the metasurface-based applications with more powerful accomplishments such as single-photon source with high efficiency and large fidelity^14^, nanolasing with great chirality^15^, information storage with high capacity and safety^16–19^, etc.

Orbital angular momentum (OAM) is a special DoF of optical fields. Its formula is given by *e*^*i*(*lθ*)^, where *l* and *θ* represent its topological charge and azimuth angle, respectively. Since its helical wavefronts have a physically unbounded set of orthogonal modes (i.e., infinite values of *l* in theory), the control of OAM has been widely regarded as the key to future information processing (including transmission, computation, storage, etc.) with ultra-high capacity and speed^20–23^. Therefore, in the realm of nanophotonics and integrated optics, great efforts have been devoted to the study of manipulating the DoF of OAM by metasurfaces^24–27^.

Generally, the metasurface-based OAM manipulation studies can be divided into two main directions. The first and most common research direction is the generation of OAM beams (i.e., incident light is plane wave). By employing principles such as Dammann grating and Bessel function, metasurfaces can generate multiple OAM beams or perfect vortex beams^28–30^, which has led to numerous advances in optical and quantum communication, biometric image recognition, and optical tweezers, greatly expanding the applications and development scope of modern optics. The other direction is the control of OAM beam^31–33^. This is a field with great challenges since the helical wavefronts have non-uniform phase distributions for different orthogonal modes compared with the plane wave. As a result, the overlap of multiple OAM beams in real space increases the difficulty of metasurface design, and calibrating the optical singularity of incident OAM beams with the metasurface also adds to experimental complexity. Till now, the manipulation of OAM multiplexed with other optical DoFs is still challenging, which notably limits the future development of nanophotonics and integrated optical devices.

Herein, we propose the idea of multiplexed coherent pixel (MCP) metasurfaces, which not only realize the full control of amplitude and phase with the incident lights of plane and OAM waves, but also achieve the multiplexed manipulation of OAM and wavelength, which consists of two printing images and eight holographic images based on a single-layer metasurface. Moreover, further design improvements will offer the potential for additional channels induced by other wavelengths and more OAM modes. We firmly believe that this work not only enriches the means of light manipulation but also provides valuable insights for the future design of high-capacity photonic devices.

## Results

### Illustration of the multiplexed manipulation of OAM and wavelength

The development of metasurfaces has mainly relied on expanding the metasurface ability of controlling optical DoFs^34–38^. To be specific, with tremendous endeavor devoted to the field of metasurfaces, a breakthrough has been achieved wherein multiple OAM channels with different topological charges *l* have been successfully applied for holographic information storage by a phase-only metasurface^33^, enabling the unprecedented data density with negligible crosstalk. However, in order to meet the future demand for nanophotonic devices for optical field control, more improvements are still required. For example, with the purpose of increasing the capacity, speed, and accuracy of optical information processing in one metasurface chip, merely controlling the OAM degree of freedom and manipulating the phase distribution are insufficient. Hence new approaches for OAM control multiplexed with other optical DoFs, as well as simultaneous control of amplitude and phase, are urgently in need. In addition, it is also important to obtain more optimized strategies for metasurface design, so as to reduce fabrication difficulties.

**Fig. 1:**
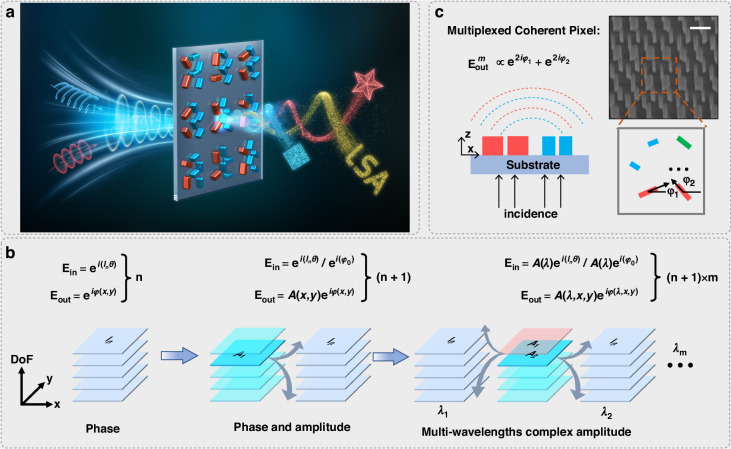
Illustration of the design of MCP metasurface and the evolution of OAM manipulation based on metasurfaces. **a** Printing and holographic images are shown through MCP metasurface when incident beams are plane and OAM waves with specific wavelengths. **b** The evolution of OAM manipulation based on metasurfaces can be divided into three parts: phase-only metasurface, complex-amplitude metasurface, and multi-wavelengths complex-amplitude metasurface. n and m, respectively, represent the number of topological charges and wavelengths. The data dimensions increase with the evolution path. **c** The illustration of the MCP metasurfaces, including the SEM image and diagram of MCP. The scale bar represents 500 nm and the blocks with different colors represent the nanopillars with different sizes

In order to meet the above-mentioned requirements for metasurface-based OAM manipulation, we propose the idea of MCP metasurface. As shown in Fig. 1a, the MCP metasurfaces can respond to both plane wave and OAM beam with different wavelengths, and respectively generate printing and hologram images, realizing the multiplexed manipulation of OAM and wavelength. For the coherent pixel, it usually consists of several nanopillars of the same size. Since the transmitted optical field of circularly polarized wave is provided by all the nanopillars within one coherent pixel, the metasurfaces based on coherent pixels can independently manipulate the amplitude and phase by easily adjusting the rotation angle of each nanopillar^39^. Therefore, by employing the coherent pixels, it can expand the scope of OAM manipulation from phase alone to complex-amplitude, increasing the dimension of information processing from n to n + 1 (Fig. 1b, from left to middle, n is the number of topological charges). Furthermore, if nanopillars with more sizes are involved, the coherent pixel will be promoted to the multiplexed coherent pixel (Fig. 1c), which can independently control the amplitude and phase with different wavelengths under the incident cases of both OAM and plane waves. This improvement can achieve a data dimension of m × (n + 1) (m represents the number of wavelengths), significantly enhancing the metasurface capability in information processing (Fig. 1b, from middle to right). In the following parts, in order to clearly elaborate the implementation of MCP, we will theoretically and experimentally demonstrate the multiplexed manipulation of 8 OAM modes in 2 wavelengths.

### Design of the multiplexed coherent pixel for OAM and wavelength multiplexing

There are three steps to complete the design of MCP metasurfaces. (1) Identifying the optimized geometric parameters of nanopillars, so as to ensure the ability to efficiently respond to multiple independent wavelengths without crosstalk. (2) Establishing the relationship between the rotation angle of nanopillars and the complex-amplitude of the transmitted wave, enabling the decoupling and independently controlling the phase and amplitude. (3) By employing relevant algorithms, the complex-amplitude components of the reconstructed signals are determined, and the specific arrangement of the nanopillars are confirmed.

**Fig. 2:**
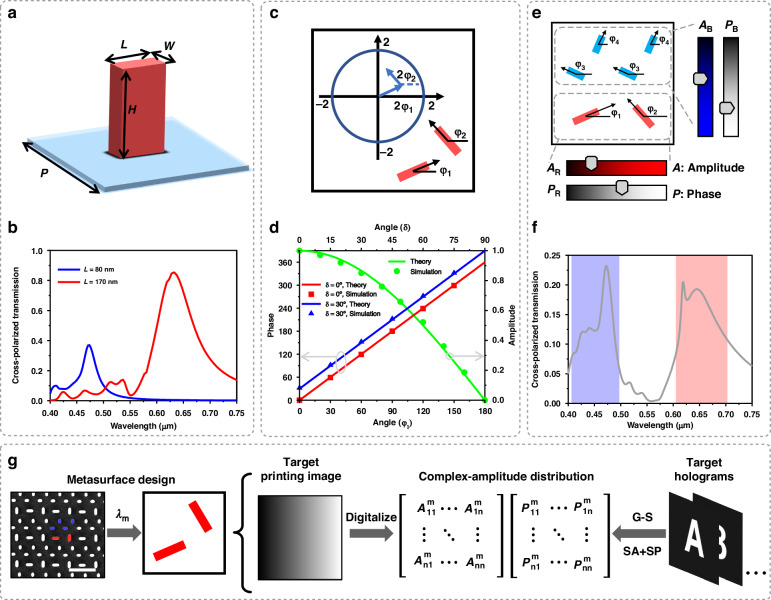
Design of c-silicon nanopillars of the MCP metasurface and continuously tuning of amplitude and phase. **a** Schematic diagram of a single c-silicon nanopillar (red) with width *W*, length *L*, and height *H* standing on the fused SiO_2_ substrate (blue). **b** Simulation results of a periodic single unit with *L* = 80 nm (blue line) and 170 nm (red line), respectively. The width, height, and period are fixed (*W* = 40 nm, *H* = 600 nm, *P* = 400 nm). The cross-polarized transmission, i.e., RCP/LCP conversion, is normalized. **c** The diagram of a coherent pixel is in the lower right corner, and the rotation angles of these two nanopillars are *φ*_1_ and *φ*_2_. The upper left diagram shows the transmission field in vector space, where these two vectors correspond to the functions of two nanopillars respectively. **d** The simulation results of a single coherent pixel. The green line and dots represent the results of amplitude manipulation. The blue and red show the results of phase adjustment. The period *P* is 550 nm. **e** Schematic of a typical MCP for arbitrary complex-amplitude tuning of transmitted waves. Each unit cell consists of six c-silicon nanopillars with rotation angles from *φ*_1_ to *φ*_4_, relative to the horizontal axis. The period *P* is 800 nm. **f** The simulated cross-polarized transmission of the structure which is shown in Fig. 2e, and the rotation angle of six nanopillars is 0°. **g** Flow chart of designing MCP metasurface. The left side is a scanning electron microscopy (SEM) image of a partial region of the fabricated c-silicon metasurface, and the colors we add (red and blue) correspond to the working wavelengths. The scale bar of this SEM image is 800 nm. SA sample array, SP spiral phase, G–S optimized G–S algorithm

In the first step, the simulated unit is a crystal silicon (c-silicon) nanopillar placed on a fused SiO_2_ substrate (Fig. 2a), and a circularly polarized beam is incident from the substrate to the nanopillar. By analyzing the transmission magnitudes of a cross-polarized beam, the length *L*, height *H*, and width *W* of nanopillars can be optimized. To be specific, our simulation results show that *H* mainly affects the transmission magnitude, but with too large *H*, the improvement in transmission magnitude caused by larger *H* is no longer obvious (Supplementary Fig. S1a). Therefore, in order to trade off the difficulty of metasurface fabrication and transmission magnitude, we have set *H* as 600 nm. On the other hand, *W* can affect the position of resonant peaks, and increasing *W* will lead to the emergence of a new resonant peak at a shorter wavelength (Supplementary Fig. S1b). Hence, to minimize the interference between two different wavelengths within our fabrication ability, we set the *W* to 40 nm. In addition, it is found that larger *L* can lead to a remarkable redshift of the transmission peak (Supplementary Fig. S1c). According to this fact, and considering that we need to design the geometric parameters of the nanopillars within an MCP to ensure minimal crosstalk between two different wavelengths, two values of *L* (80 and 170 nm) are selected for blue (wavelength of 473 nm) and red beams (wavelength of 633 nm) respectively (Fig. 2b), making two nanopillars exhibit sharp and obvious transmission peaks at our designed wavelengths. Other details of the numerical simulations are explained in the “Materials and Methods” section. The simulated results with other materials are shown in Fig. S2 (Supplementary Information).

Next, we turn to step 2 to ensure the ability of our MCP metasurface to independently control the phase and amplitude of the transmitted optical field. For conventional metasurfaces, which only have one nanopillar in one unit pixel, realizing the amplitude control of circularly polarized light often requires changing the shape of nanopillars (also see Supplementary Fig. S1), which is not only difficult to obtain arbitrary amplitude value, but also holds large fabrication cost. Accordingly, if the geometry of nanopillars remains unchanged, only the phase of the cross-polarized wave can be manipulated with a 2*φ* phase delay (Pancharatnam−Berry Phase), where *φ* is the rotation angle of the nanopillar on the x-y plane. Therefore, to achieve the intention of simultaneously manipulating phase and amplitude in step 2, we will utilize the coherent pixel method, where each unit contains two nanopillars with the same size for controlling light of one wavelength.

To be specific, here is the transmission coefficient of the cross-polarized wave through the coherent pixel cell when the incident beam is the other circularly polarized wave:

1 $$E\propto {e}^{2i{\varphi }_{1}}+{e}^{2i{\varphi }_{2}}=2\,cos\, \delta {e}^{i(2{\varphi }_{1}+\delta )}$$

where *φ*_1_ and *φ*_2_ are the rotation angles of the nanopillars relative to the x-axis, and *δ* refers to the rotation angle difference between the two nanopillars, so *δ* = *φ*_2_-*φ*_1_. Detailed deduction of this expression is given in Supplementary Note S1. Therefore, the amplitude and phase are manipulated by cos*δ* and (2*φ*_1_ + *δ*), respectively. As shown in Fig. 2c, in vector space combining these two nanopillars, which are presented as two vectors with rotation 2*φ*_1_ and 2*φ*_2_, the final vector can reach any point of the whole space. This means that we have decoupled the amplitude and phase without additional shape design for nanopillars. To verify the feasibility of this method, we simulate a periodic structure which contains two nanopillars (*L* = 80 nm, *W* = 40 nm, *H* = 600 nm). The simulation results (dots) are obtained by commercial software, which enables the analysis of the phase and amplitude of light transmitted through the two nanopillars (Fig. 2d). The simulated results agree well with the theoretical ones (lines), confirming the feasibility of controlling complex-amplitude by MCPs.

**Fig. 3:**
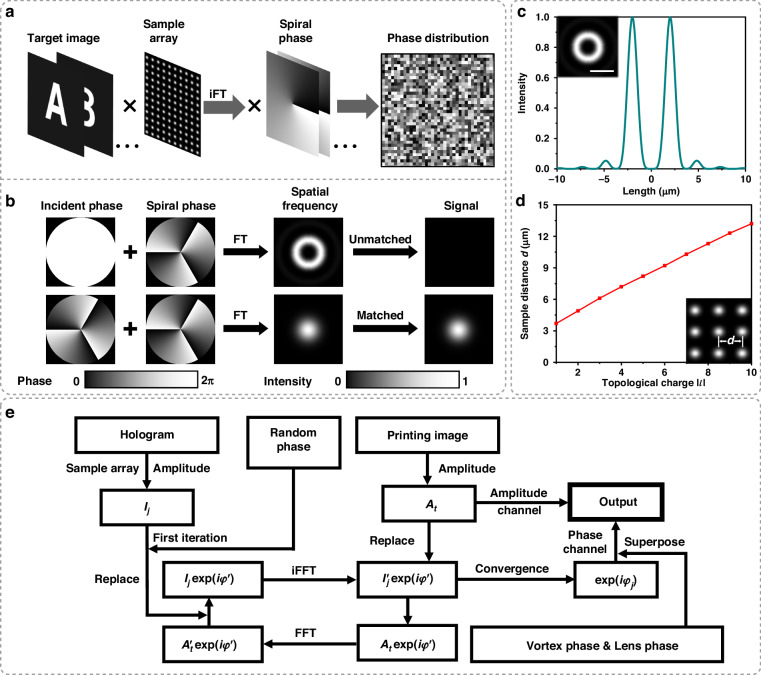
The design of obtaining the complex-amplitude distribution. **a** Design strategy of OAM holograms with multiple topological charges *l*. iFT represents the inverse Fourier transform. **b** Design principle of the spiral phase for the multiplexing of multiple topological charge *l*. The topological charges for the incident wavefront and spiral phase plane are *l*_in_ and *l*_sp_ respectively. FT represents the Fourier transform. **c** Intensity distribution of OAM wave in the focal plane. The OAM wave (*λ* = 633 nm) with topological charge (*l* = 3) is incident on a lens (diameter is 720 μm and focus distance is 1.36 mm). The subfigure shows the intensity distributions on the 2D focal plane, and the scale bar represents 4 µm. **d** Numerical characterization of sample distance as a function of topological charge |*l*|. The subfigure illustrates the definition of the sample distance *d*. **e** Flow chart of the complex-amplitude design for the integration of printing images and OAM holograms. ${I}_{j}$ and ${A}_{t}$ are the amplitudes of the OAM holograms and printing images, respectively. FFT and iFFT represent the fast Fourier transform and inverse fast Fourier transform, respectively

Based on the discussion above, the basic and typical MCP can be designed properly. As demonstrated in Fig. 2b, the cross-polarized transmission of blue nanopillar (*L* = 80 nm) is lower than red nanopillar (*L* = 170 nm). In order to ensure that the intensity of the blue signal is comparable to the red signal, there are two identical coherent pixel units of blue signal in one MCP. The final MCP contains six nanopillars, where four nanopillars of blue beam are arranged diagonally in the first and second quadrants, and two nanopillars of red beam are placed in the center of the third and fourth quadrants (Fig. 2e). To mitigate the interactions between these nanopillars, the period of this MCP is set as 800 nm. By simulating this periodic structure, the result of conversion efficiency meets the design requirements (Fig. 2f). After the two steps, we can confirm that the MCP block can achieve the manipulation of arbitrary complex-amplitude (phase 0-2π, amplitude 0-1) for incident lights of two wavelengths (473 and 633 nm). Hence, for the next step, we only need to obtain the specific arrangement of each MCP and the final design of our metasurfaces can be determined (Fig. 2g). But, to obtain the phase distributions for multiple holograms generated by different OAM modes (i.e., different *l* for different holograms), together with the requirement of integrating printing and hologram information, systematical considerations should be conducted. Hence, we will elaborate on this process, especially in the following part of Fig. 3.

### The process of obtaining complex-amplitude distribution

Figure 3a illustrates the basic idea of obtaining phase distributions of different hologram images generated by OAM beams with different modes. The target holographic images are first sampled by a 2D Dirac comb, namely a sampling array, and then superimposed on the spiral phase after the inverse Fourier transform. At last, by superposing these results, we obtain the phase distribution, which exhibits distinct responses to incident OAM beams with different *l*, thereby achieving parallel processing of multiple OAM information channels.

The selections of spiral phase and sampling array are crucial in the above strategy, where the spiral phase transforms conventional signals into OAM signals, namely imparts the property of OAM (unbounded and orthogonal modes) to our phase plane. An appropriate sampling array can maintain the separation of OAM signals in momentum space, thereby preserving the functionality of the spiral phase and avoiding crosstalk between different information channels. Next, a detailed exposition of the function of the spiral phase is provided in Fig. 3b.When a plane wave is incident to the spiral phase plane (upper part of Fig. 3b), the reconstructed signal after the Fourier transform reveals doughnut-shaped intensity distribution. If the incident wavefront and spiral phase plane have the same spiral phase distribution but opposite topological charge (lower part of Fig. 3b), the result will be a spot. When an algorithm like a low-pass filter is utilized, only the signal with matched topological charge (*l*_in_ = -*l*_sp_) can pass through. Similarly, the applicability of this method can be extended from individual spiral phase to complex-amplitude plane of multiple holograms (Supplementary Note S2). In this way, the foundation for our subsequent integration of printing and hologram images can be established.

Next, we need to carefully select an appropriate sample array to avoid the overlap of OAM signals in momentum space. Hence, we analyze the distribution of OAM signals in momentum space, and its intensity profile can be numerically described by a formula^40^ (Supplementary Note S3). When a beam with a wavelength of 633 nm is incident, the intensity profile of the electric field is given in Fig. 3c. In the convolution process (Supplementary Note S2), when these rings are overlapping, the fidelity of reconstructed holograms will be affected. Since the region with low intensity has a negligible impact on the holograms, we define the region where the intensity is greater than 0.1 as non-overlapping areas. Therefore, the interval of the sample array can be set according to the above standard. For example, the sample distance in Fig. 3c is about 6.1 μm. Figure 3d shows the interval of the sample array as a function of topological charge $\left|l\right|$ under the above condition. The distance with other wavelengths is shown in Supplementary Note S3.

After completing the design of the OAM hologram, we aim to integrate the corresponding phase components with the amplitudes of printing images. Therefore, we develop a modified G–S algorithm, and a flow chart of this strategy is depicted in Fig. 3e. First, the amplitudes ${I}_{j}$ (*j* = 1, 2, 3…) of holograms, which are processed by sample array, are extracted. Next, after a random phase is added to ${I}_{j}$, these complex amplitudes enter a loop about the fast Fourier transform (FFT) and inverse fast Fourier transform (iFFT). In this loop, amplitudes ${A}_{t}^{{\prime} }$ and$\,{I}_{j}^{{\prime} }$ are constantly replaced by ${I}_{j}$ and amplitudes of printing images ${A}_{t}$ (*t* presents different wavelengths) respectively. When this loop meets the convergence condition, the phase terms $\exp (i{\varphi }_{j})$ are extracted. Then specific vortex phases are added to $\exp (i{\varphi }_{j})$. After that, these phase components corresponding to a certain wavelength are added up and superposed by a lens phase. Finally, the new phase terms and amplitudes ${A}_{t}$ are integrated as complex amplitudes, which can be manipulated by our MCP method. The experimental demonstration of the feasibility of the above-mentioned theoretical consideration is presented in Supplementary Fig. S3.

### Experimental demonstration of the multiplexed manipulation of OAM and wavelength

**Fig. 4:**
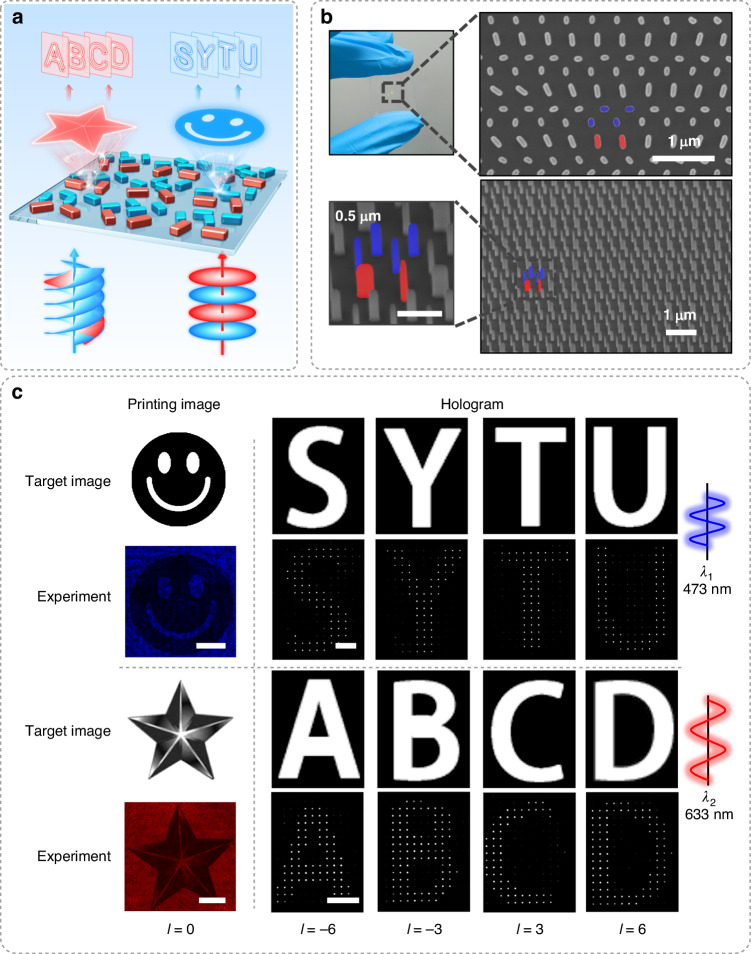
Experimental demonstration of the MCP metasurface for the multiplexed manipulation of OAM and wavelength. **a** Plane and OAM waves propagate normally into the MCP metasurface with two wavelengths (red: 633 nm, blue: 473 nm). **b** Upper: The metasurface and its SEM image. Lower: SEM images of the sample (φ_1_ = φ_3_ = 0° and φ_2_ = φ_4_ = 90°). The colors we add (red and blue) correspond to the working wavelengths. **c** Printing and holographic images are reconstructed by one MCP metasurface. Left: a binary image and a grayscale image. The scale bar is 100 µm. Right: different Latin letters reconstructed by incident OAM waves with different topological charges (*l* = −6, −3, 3, 6) and wavelengths. The scale bar is 70 µm. For clear display, the printing images show the intensity corresponding to the wavelength, and the holograms only show the intensity with white

Next, we experimentally fabricate the MCP metasurfaces which can realize the integration of printing and holographic images. As illustrated by Fig. 4a, our MCP metasurface can respond to plane and OAM lights with different wavelengths simultaneously, presenting printing images such as the smiling face and pentagram, as well as the holographic Latin letters. The upper SEM image (Fig. 4b) represents the employed metasurface with a resolution of 1200 × 1200, which realizes the integration of printing and holographic images. Therefore, all the rotation angles of nanopillars are different. The lower SEM image shows the sample of a specific pixel with the outputting amplitude intensity of zero and phase of π/2 (i.e., φ_1_ = φ_3_ = 0° and φ_2_ = φ_4_ = 90°). The rotation angles of 0° and 90° are typical cases in our metasurfaces, and we choose to show them also because such SEM image is of benefit to observing the shape of the prepared nanopillars. From the SEM images, we can see that these pillars meet our design parameters (*L* = 170 and 80 nm, *W* = 40 nm) and have straight sidewalls. It is worth noting that the straight sidewalls are essential for both printing images and holograms. For instance, the concave sidewalls result in a broadened width of transmission profile and a decrease of conversion peak (Supplementary Fig. S4).

The experimental measurement results of ten image integrations are given in Fig. 4c, where two amplitude channels support two printing images, and two-phase channels present eight OAM holographic images. When a plane wave or OAM wave is incident, the related image appears on the corresponding plane. The left side of Fig. 4c illustrates that the metasurface can achieve a good presentation of these printing images on the metasurface plane (z = 0, z stands for transmission distance). The blue plane wave (*λ* = 473 nm) can reconstruct a binary smile face. Similarly, a five-pointed star with grayscale shows a stereoscopic impression, when a red plane wave (*λ* = 633 nm) is incident. By comparing these two images, we can see that there is almost no crosstalk, which means that our metasurface has the capability of multichannel transmission.

When the source is switched to the OAM wave, the holograms will be reconstructed on the Fresnel diffraction planes (z = 2.9 mm for the wavelength of 473 nm, z = 1.6 mm for the wavelength of 633 nm). OAM waves with different topological charges (*l* = −6, −3, 3, 6) reconstruct different Latin letters (473 nm: S, Y, T, U; 633 nm: A, B, C, D). So far, we have accomplished large-capacity data storage and encryption in an easy-fabricated structure which has not been realized prior to this. In addition to the fabrication imperfections and measurement errors, the factor that affects the quality of these holograms is the interval between topological charges. When the interval changes from 3 (*l* = −6, −3, 3, 6) to 1 (*l* = −2, −1, 1, 2), the quality of the holograms can be deteriorated (Supplementary Fig. S5). The main cause is the crosstalk between the topological charges of the same wavelength. The holograms with other intervals are shown in Fig. S6 (Supplementary Information). In these MCP metasurfaces, the optical efficiencies of images can be ~23 and ~5.4% in theory and experiments, respectively. The difference between experimental and theoretical results is caused by the existence of those MCPs with purposely manipulated amplitude (i.e., output the specific and required amplitude value rather than the maximum), fabrication process error, and the loss of optical components such as polarizer and wave plate. It should be mentioned that our efficiency results are comparable with previous works^41–43^, which show efficiencies of ~25 and 4–10% in experiments and theory, respectively. In order to achieve the maximum increase in the efficiency of MCP metasurfaces, the spacing between adjacent MCPs is set to zero. Due to the reason that the variation of rotation angles (for example, the difference of *φ*_1_ between adjacent MCPs) in most regions of the entire metasurfaces is delicately designed to be continuous and gradual, the coupling between adjacent MCPs becomes small enough for generating high-quality images as shown in Fig. 4c. Herein, we reduce the crosstalk by structure optimization and selecting suitable sample array. However, it is also noticed that recent work has incorporated crosstalk into the design process^44^, which could enlighten new methods to further enhance the performance of metasurfaces.

The integration of holographic and printing images holds significance in areas such as data storage, information security, advanced display, etc.^45–47^. Different from prior works that solely employ plane waves for integrating printing and hologram images, our MCP metasurfaces enable the OAM beams as a novel DoF, thereby achieving much higher capacity in one metasurface. In previous works which can achieve the manipulation of arbitrary complex-amplitude^16,39,41,48–50^, a benchmark work about integrating holographic and printing images has reported the ratio of basic pixel size to channel number to be 0.135 μm^2^ channel^-1^ (i.e., pixel size with 0.405 μm^2^ for three channels and pixel size with 16 μm^2^ for six channels)^41^. However, in this work, due to the employment of OAM beams, we can reduce such ratio to 0.064 μm^2^ channel^−1^ (i.e., pixel size with 0.64 μm^2^ for ten channels), which indicates that we can at least double the integrating degree and storing capacity. Moreover, for the printing image, we can obtain both the grayscale and binary printing images, because our MCP method enables continuous manipulation of the complex-amplitude of transmitted optical field. It is well-known that grayscale images, in comparison to binary images, are more vivid and stereoscopic, which indicates greater information density and broader application prospects. It should be mentioned that the reason for digitized holograms is that we want to realize the multiplexed control of multiple OAM channels in different wavelengths, rather than that the subwavelength nanopillars (can also be called metasurface lattices) or the spacing between nanopillars are not small enough. One can find that the grayscale and binary printing images are continuous and smooth (Fig. 4c, left column), which demonstrates our metasurfaces are well-designed and fabricated. Based on the above discussions, one can understand that our MCP metasurfaces have greatly improved the metasurface function of storing printing and hologram information based on realizing the multiplexed control of wavelengths and topological charges of OAM beams.

## Discussion

With the demand for processing information with ultra-high speed and capacity in our information era, increasing the ability of manipulating the optical DoF of OAM is becoming an important and growing research focus in fields varying from integrated optics, and information communication, to quantum technology, on-chip nanophotonics, etc. However, a disappointing fact is that the current manipulation of OAM mainly remains in the single-DoF level, indicating that there are few effective ways to simultaneously control the OAM and other optical DoFs. Therefore, in this work, we use the MCP metasurfaces to realize the multiplexed manipulation of OAM with wavelength, and our main advancements are as follows.

Firstly, the MCP metasurface can achieve arbitrary complex-amplitude control of transmitted lights, and it also expands the type of incident light which metasurface can simultaneously respond from OAM or plane wave to both of them. Secondly, with the ability of realizing multiplexed manipulation of OAM and wavelength, we achieve ten information recording channels for a single-layer metasurface. When these channels are used to integrate printing and hologram images, we can reduce the ratio of basic pixel size to channel number to be as small as 0.064 μm^2^ channel^-1^, which is a new record for the integration of printing and hologram images by a single-layer metasurface with arbitrary complex-amplitude control. Thirdly, it is noteworthy that the information capability of such metasurfaces can be further enhanced by incorporating other wavelengths (integrating other nanopillars with different parameters in an MCP) and more topological charges. In addition, the function of our MCP metasurface can be further explored, and a super-encryption method can be realized, which is composed of two encryption strategies in one metasurface (Supplementary Fig. S7).

**Fig. 5:**
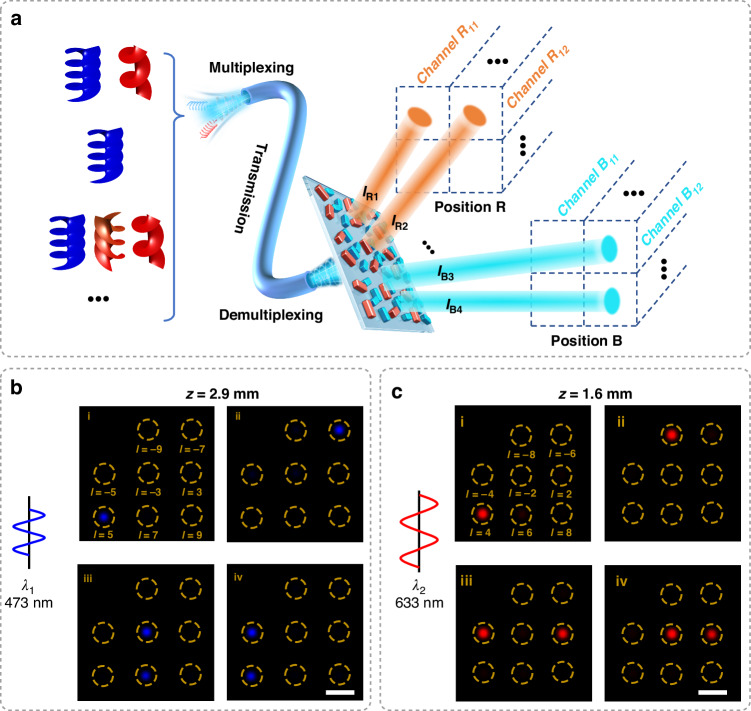
Experimental demonstration of the MCP demultiplexer. **a** Illustration of the demultiplexer. **b** Experimental results of demultiplexing with a wavelength of 473 nm. The scale bar is 30 μm. **c** Experimental results of demultiplexing with a wavelength of 633 nm. The scale bar is 20 μm

Apart from the function of information storage and encryption, the application of MCP metasurfaces can be further explored due to the advantage of controlling the DoFs of OAM multiplexed with wavelength. As shown in Fig. 5, we find that our MCP metasurfaces can be applied as the nanophotonic device of an OAM demultiplexer with 16 channels working under two wavelengths with 8 topological charges. Due to the widespread employments of OAM beams in edge imaging, quantum communication, and optical tweezers, the OAM demultiplexer, which serves as a pivotal component for multichannel information transmission, has gained growing attention due to its ability to process multiple incident signals simultaneously. With the introduction of metasurfaces which brings about advantages of customization and miniaturization, the metasurface-based OAM demultiplexer has exhibited remarkable advances in optical chip, biometric sensing, information processing, etc. However, the existing demultiplexers cannot achieve the multiplexing with different OAM modes and wavelengths, which leads to the limit in the number of different channels and the loss of photonic advantages of multiple DoFs. However, our MCP metasurfaces can spatially separate OAM beams of different *l* and different wavelengths simultaneously.

As shown in Fig. 5a, by designing this MCP metasurface, signals of different wavelengths can be diffracted onto different Fresnel diffraction planes. Furthermore, different OAM modes *l* can be demultiplexed to different positions on the Fresnel diffraction planes, ultimately resulting in distinct information channels. Figure 5b, c experimentally demonstrates this functionality. Firstly, when only one kind of OAM beam is incident, OAM signals with different wavelengths and different *l* can be routed to different pre-designed positions (figure i and ii in Figs. 5b, c). Secondly, when compound OAM signals are incident, it is found that these signals can be distributed to pre-designed positions without crosstalk, which confirms the robustness in routing capability of our MCP metasurfaces. In fact, this metasurface can simultaneously process more topological charges. However, due to limited experimental conditions, we could only demonstrate the demultiplexing of two topological charges. We believe our metasurfaces provide insights for the integration of photonic chips and applications in optical communications in the future. More information about the application of OAM demultiplexer is also given in Fig. S8 (Supplementary Information).

Our work not only demonstrates the MCP method for metasurface design, but also raises the OAM manipulation from single-DoF level to multiple-DoF level, which will significantly increase the data capacity and routing channel of metasurfaces. We believe those findings can be extended and generalized easily to next-generation encryption techniques and multiplexed optical devices.

## Materials and methods

### Numerical simulation and calculation

The numerical simulation is calculated by commercial finite-difference time-domain (FDTD) software (FDTD solutions, Lumerical Inc.). The boundary conditions in horizontal (*x*- and *y*-) and vertical (*z*-) directions are set as a periodic boundary condition and perfect matched layer, respectively. To ensure that the result of the simulation is acceptable, the mesh accuracy and simulation time are level 6 and 10,000 fs. The refractive index of the SiO_2_ substrate is set as 1.45, which remains nearly constant in the visible spectrum. The dielectric constants of c-silicon and amorphous silicon are sourced from Palik^51^ and Pierce^52^, respectively. The NOA61 Layer is negligible in the simulation because the refractive index of NOA61 (about 1.55) is similar to that of SiO_2_ and the thickness of NOA61 is much smaller than that of the SiO_2_ layer. The program of metasurface design, image digitization, signal filtering, and drawing are generated by MATLAB software.

### Transfer and fabrication of c-silicon metasurface

As shown in Supplementary Fig. S9, the flow chart represents the entire metasurface fabrication process. (1) A SOI wafer is etched by ICP (HBr), and the thickness of the upper c-silicon layer is reduced from 1000 to 600 nm. (2) A 500-nm-thick SiO_2_ is deposited on top of the SOI wafer through an inductively coupled plasma chemical vapor deposition (ICP-CVD) process. (3) After adhesive NOA61 is spun onto it, the processed SOI wafer is subsequently inverted onto a SiO_2_ substrate (thickness 1 mm). Next, the sample is subjected to UV light exposure for 5 h, followed by a 4-day baking process at a temperature of 50 °C. (4) The excess Si is removed by polish and ICP etching (SF_6_). (5) The protective layer (500 nm SiO_2_) is removed using 10% HF acid for about 8 min. (6) 140 nm HSQ is spin-coated on the sample with 4000 rpm, before baked for 3 min at 93 °C. (7) A 30 nm thickness layer of aluminum is deposited using thermal evaporation, which acts as a layer for dissipating charges. (8) The metasurface pattern is fabricated by electric beam lithography (EBL) at 2000 µC cm^−2^, 100 keV. (9) The Al and HSQ layer are removed by 5% phosphoric acid and tetramethylammonium hydroxide solutions, respectively. (10) The c-silicon metasurface is fabricated through ICP (HBr) etching. The instrument model and brand for ICP is PlasmaPro System 100ICP180, Wavetest; for EBL is EBPG5000+, Raith; and for ICP-CVD is PlasmaPro System100 ICP180-CVD, Oxford. It should be noticed that if suitable commercial wafer which is a glass substrate with a thin c-Si layer can be obtained from external sources, the fabrication steps from 1 to 5 can be simplified.

### Optical measurement

The measurement of the printing and holographic images is conducted using the optical setup illustrated in Supplementary Fig. S3. The beams (473 and 633 nm) emitted from two lasers are integrated into the same optical path through a series of mirrors. To ensure simplicity, this part of the setup is not shown in the diagram. Incident OAM waves are generated by SLM (X15213-01, HAMAMATSU Inc.). Two pairs of linear polarizers and quarter-wave plates are used to generate and filter relevant circularly polarized beams. A 4-f system is used to reduce the beam spot (*f*_1_ = 200 mm and *f*_2_ = 40 mm). A ×20/0.4 objective lens collects the scattered light emitted from the metasurface. Finally, the transmitted wave is focused by a lens (*f*_3_ = 100 mm) and imaged on a CMOS (CS135MU, Thorlabs Inc.).

### Supplementary information

Supplementary information for manuscript
