# Supplementary material for: Multiplexed manipulation of orbital angular momentum and wavelength in metasurfaces based on arbitrary complex-amplitude control
Source: Light Sci Appl. 2024 Apr 28;13:98. doi: 10.1038/s41377-024-01420-6 (PMC11055872; doi:10.1038/s41377-024-01420-6)
Supplement: Supplementary file 1 — Supplementary information for manuscript [file 41377_2024_1420_MOESM1_ESM.docx]

Supplementary Information for

**Multiplexed manipulation of orbital angular momentum and wavelength in metasurfaces based on** **arbitrary complex-amplitude control**

Guoli He^1^, Yaqin Zheng^1^, Changda Zhou^1^, Siyang Li^1^, Zhonghong Shi^1^, Yanhui Deng^1^, Zhang-Kai Zhou*^,1^

^1^State Key Laboratory of Optoelectronic Materials and Technologies, School of Physics, Sun Yat-sen University, Guangzhou 510275, China

*Email: zhouzhk@mail.sysu.edu.cn

**This file includes:**

Figure S1. The simulation result of cross-polarized transmission with the variation of nanopillars size.

Figure S2. Simulated cross-polarized transmission of amorphous silicon nanopillars.

Figure S3. The measurement and integration of printing images and OAM holograms.

Figure S4. Analysis of the influence of concave sidewalls on cross-polarized transmission.

Figure S5. Demonstration of the integration with printing images and OAM holograms.

Figure S6. Demonstration of the OAM holographic images with different topological charges (*l* = -4, -2, 2, 4).

Figure S7. Diagram of a super-encryption method.

Figure S8. Experimental results of demultiplexing for 8 and 16 channels with two wavelengths.

Figure S9. The flow chart of the entire metasurface fabrication process.

Figure S10. The flow chart of the filtering process.

Figure S11. Numerical characterization of sample distance as a function of topological charge.

Note S1. Discussion on the situations of 2 nanopillars in a pixel.

Note S2. The multiplexing of OAM holographic images.

Note S3. The calculation of sample distance.
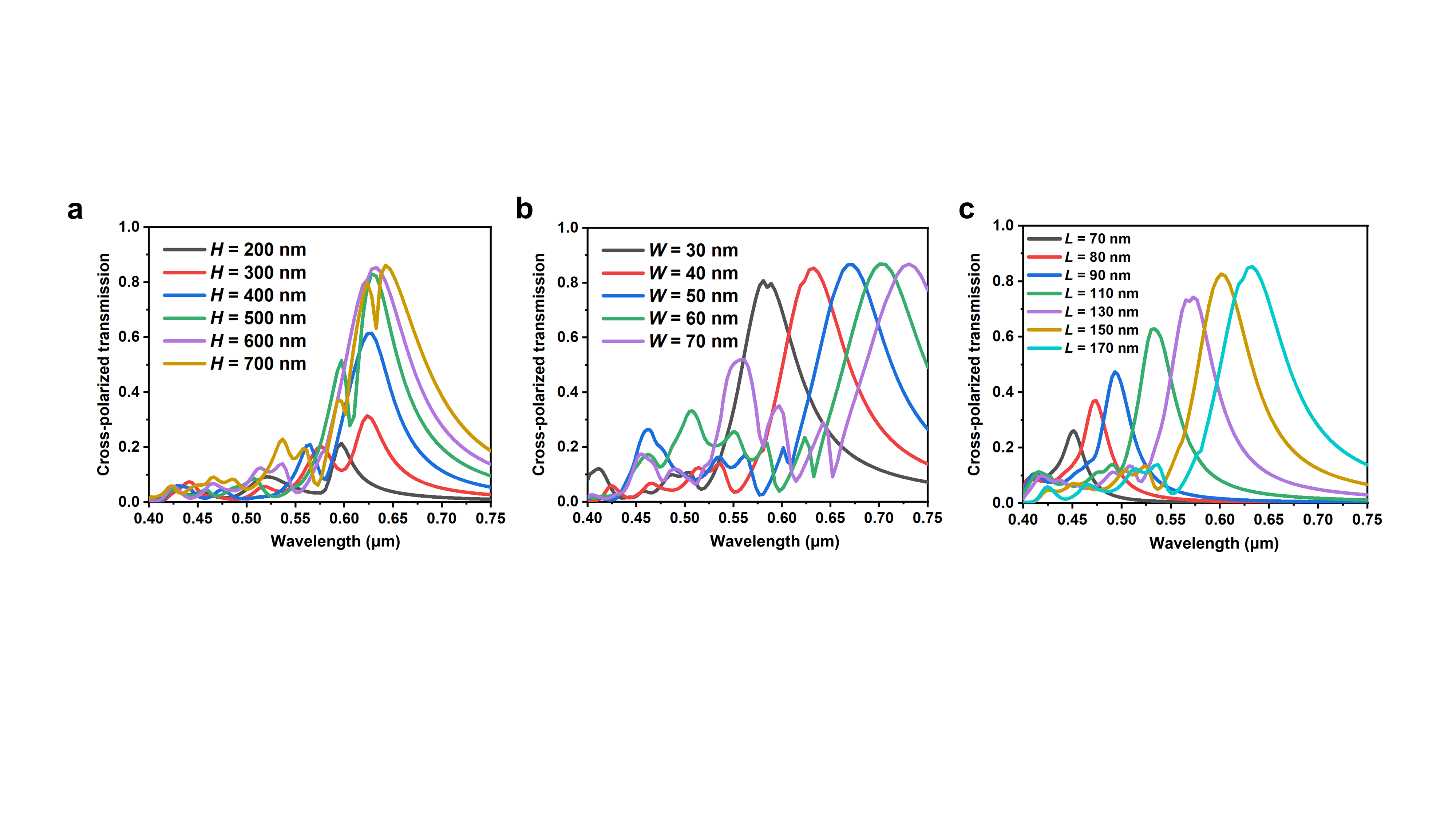


**Supplementary Fig. S1 a** Simulated transmission results of periodic single unit with different heights *H*. The width, length and period are fixed (*W* = 40 nm, *L* = 170 nm, *P* = 400 nm). The cross-polarized transmission, i.e. RCP/LCP conversion, is normalized. **b** Simulation results of the transmission with different widths *W*. The height, length and period are fixed (*H* = 600 nm, *L* = 170 nm, *P* = 400 nm). **c** Simulation results of the transmission with different lengths *L*. The height, width and period are fixed (*H* = 600 nm, *W* = 40 nm, *P* = 400 nm). If the green and blue beam are multiplexed in this platform, the minimum free spectral range is 50 nm. The height *H* of the nanopillars can affect the conversion efficiency. For the curves presenting the cases of *H* = 500, 600 and 700 nm, the difference of peak intensity is within 5%, which is a small and tolerable value. Therefore, we could conclude that to ensure the conversion efficiency, the tolerance for *H* should be ~100 nm. The size of nanopillars (i.e., length *L* and width *W*) can determine the profile of cross-polarized transmission. As our target is to achieve independent transmission for two wavelengths, the transmission peaks corresponding to the two nanopillars should not overlap. If *W* changes by 10 nm (*W* = 50 nm), the position of the transmission peak shifts by approximately 30 nm, which leads to a decrease of cross-polarized transmission by ~45%. Moreover, a new resonant peak emerges in the blue region which will lead to the crosstalk between our information channels and reduce the fidelity of the target signal. Similarly, if *L* changes by 20 nm, the position of the transmission peak also shifts by approximately 30 nm, and the cross-polarized transmission will decrease by ~25%, which will lower the conversion efficiency of target signal. Based on the above analyses, we find that the tolerances for *W* and *L* are approximately 10 nm and 20 nm, respectively. In the electrical beam lithography process, a fabrication error is ~10 nm in size, and there is almost no deviation in the rotation angles of nanopillars. For height *H*, the fabrication error of inductively coupled plasma etching is around 20 nm. In summary, our fabrication techniques can well meet our requirements in metasurfaces preparation.


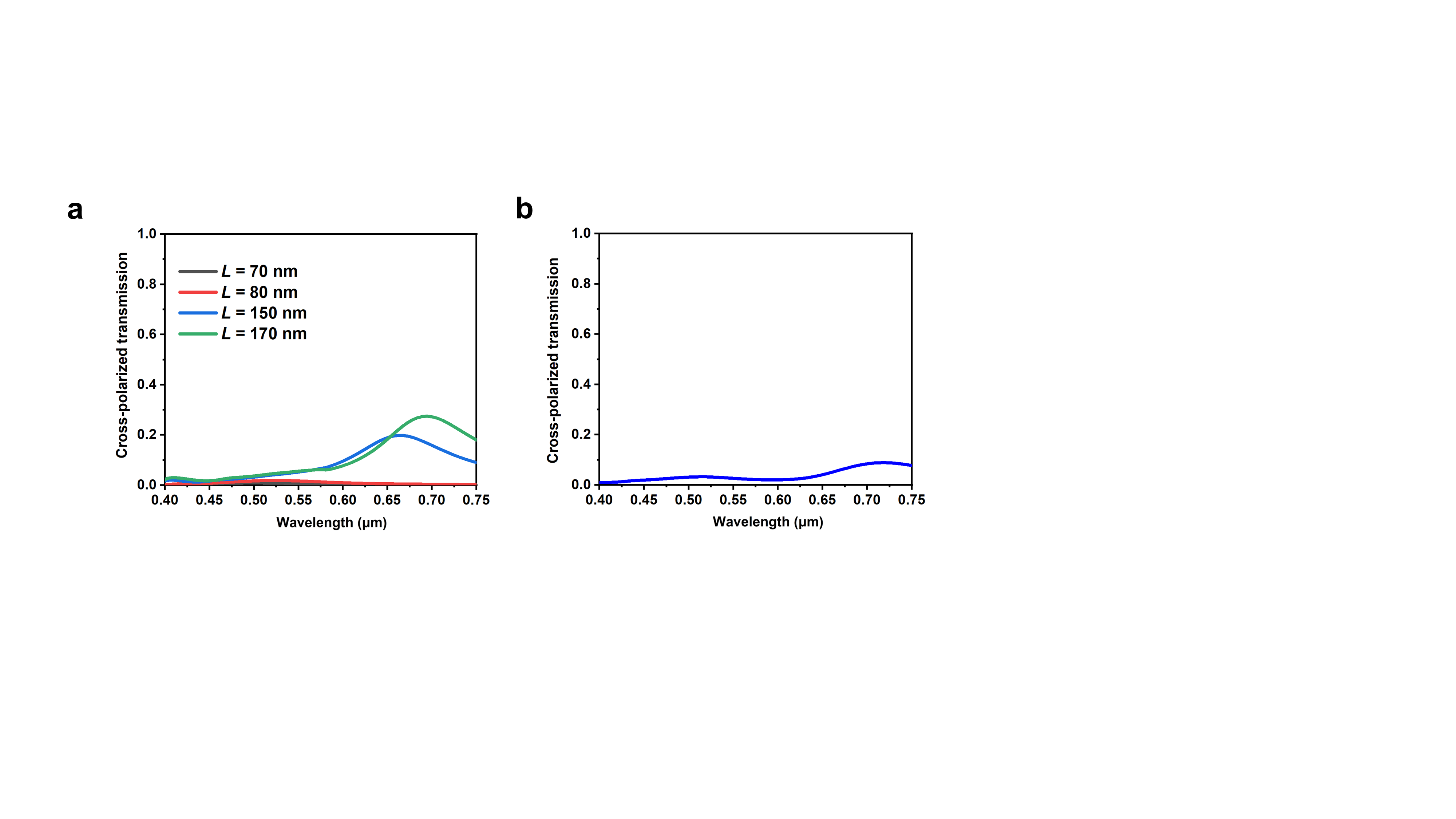


**Supplementary Fig. S2 a** Simulated transmission results of amorphous silicon nanopillars with different length *L*. The height, width and period are fixed (*H* = 600 nm, *W* = 40 nm, *P* = 400 nm). **b** The simulated cross-polarized transmission of the amorphous silicon building blocks which are shown in Fig. 2e. The rotation angle of six nanopillars is 0° relative to the horizontal axis.


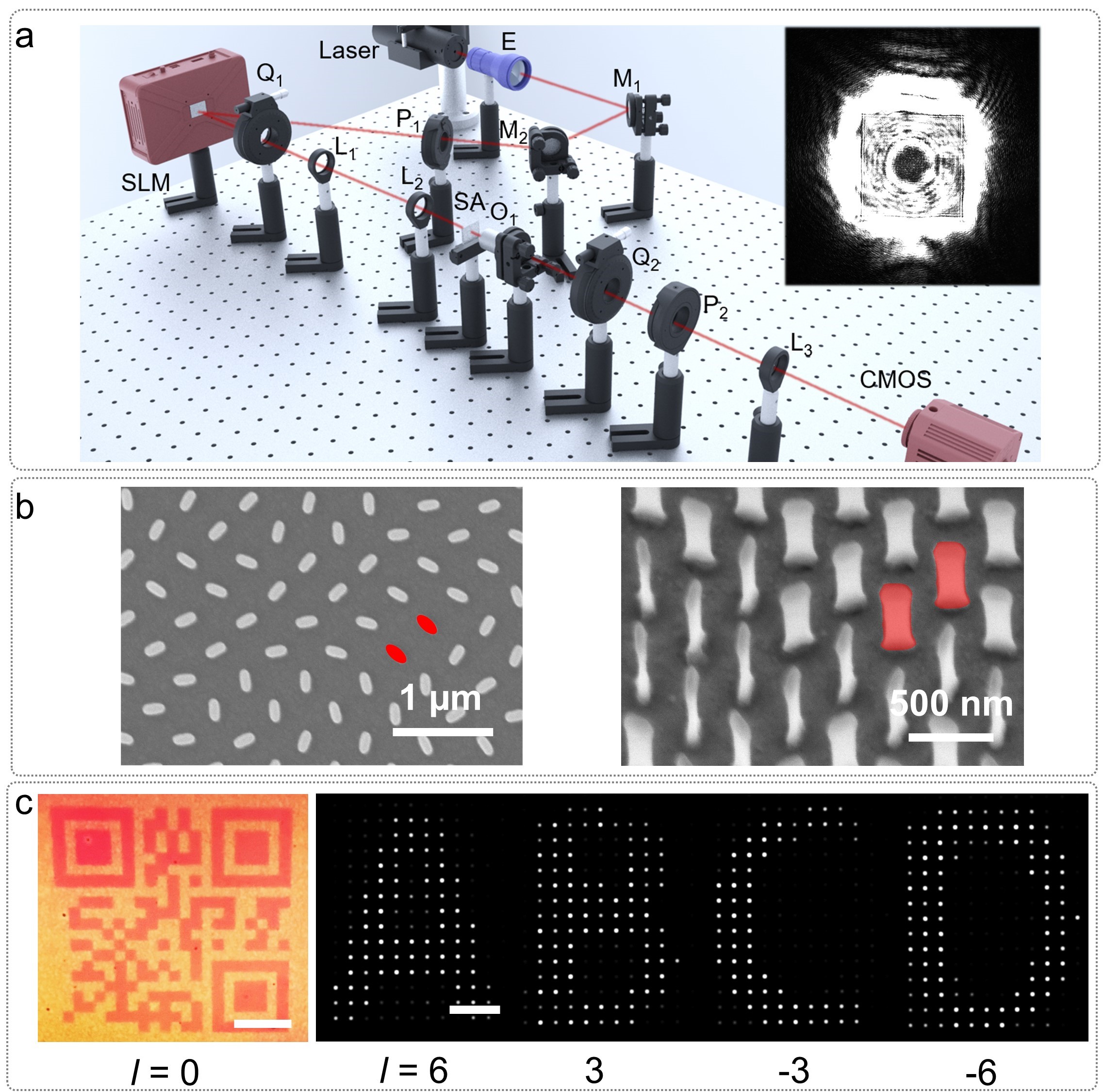


**Supplementary Fig. S3** **The measurement and integration of printing images and OAM holograms.** **a** Schematic diagram of the optical setup for measurement. E: beam expander, M: mirror, P: linear polarizer, Q: quarter-wave plate, SA: metasurface sample, L: Lens, O: Objective lens. The inset shows that the OAM beam can cover the entire metasurface, indicating the good alignment of the incident OAM beams with the metasurface. **b** SEM images of the coherent pixel metasurface and all nanopillars are identical (*L* = 160 nm, *W* = 40 nm, *H* = 600 nm), except that they rotate at different angles. The scale bars are listed in these figures respectively. **c** Printing image (the first image) of the coherent pixel metasurface and the scale bar is 70 µm. This QR code image is taken by a mobile phone, and it can be decrypted again by the phone into the password “world”. Holographic images (the last four images) correspond to different OAM beams (*l* = 6, 3, -3, -6), and the distance from the metasurface to the holographic images is 1.36 mm. The scale bar of these holograms is 70 µm.


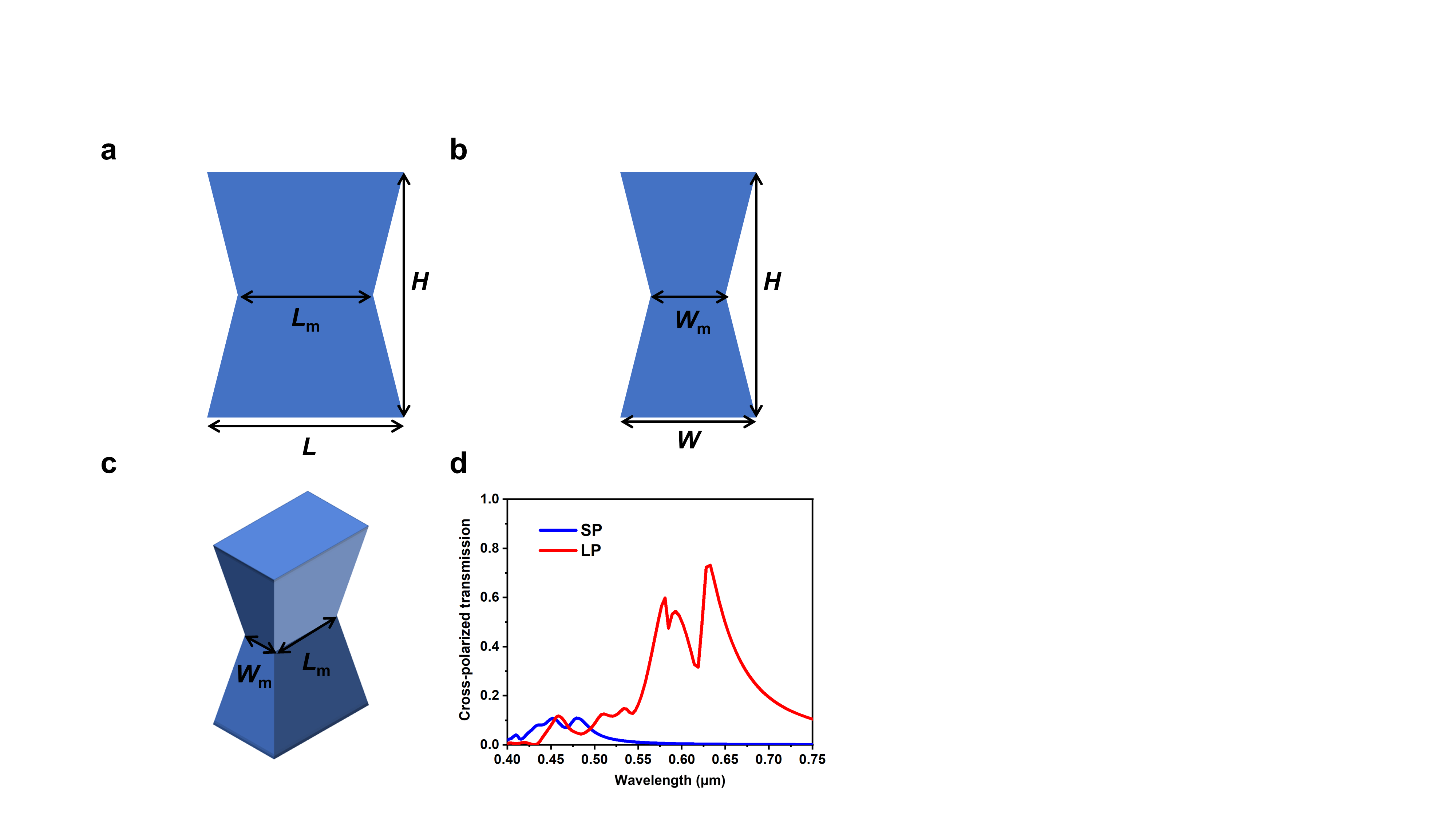


**Supplementary Fig. S4** **Analysis of the influence of concave sidewalls on cross-polarized transmission.** **a** The front view of nanopillars with concave sidewalls. **b** Side view of nanopillars with concave sidewalls. **c** Schematic diagram of this nanopillar with width *W*, length *L*, length in the middle *L*_m_, width in the middle *W*_m_ and height *H*. The *W*_m_ = 30 nm, *W* = 50 nm and *H* = 600 nm are fixed. **d** Simulated transmission results of two nanopillars with concave sidewalls (SP: *L* = 90 nm, *L*_m_ = 60 nm, LP: *L* = 180 nm *L*_m_= 150 nm).


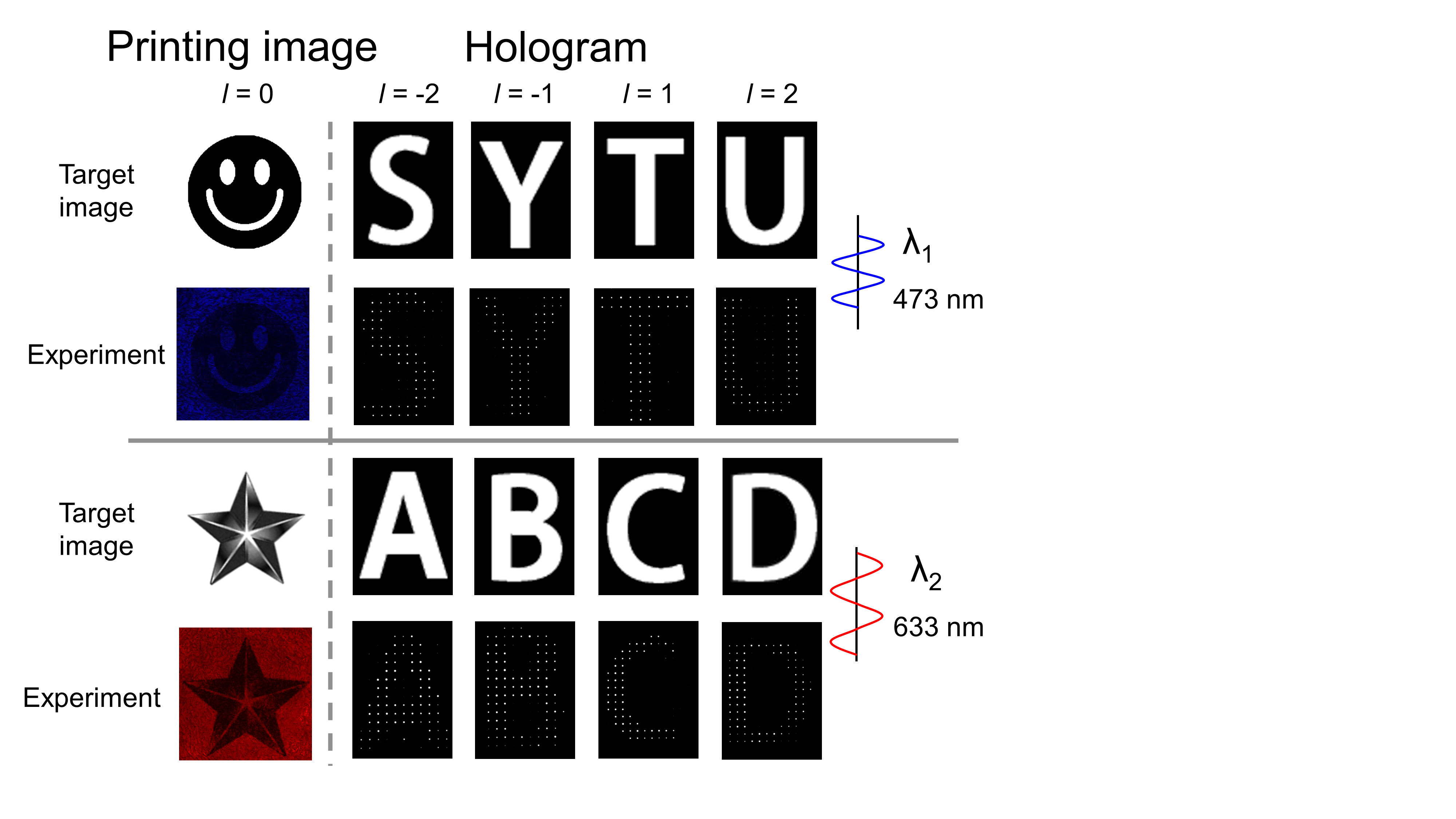


**Supplementary Fig. S5** Demonstration of the integration with printing images and OAM holograms. All parameters except pixel count (600*600) and topological charges (*l* = -2, -1, 1, 2) are consistent with the metasurface shown in Fig. 4. Plane and OAM waves propagate normally into the metasurface with two wavelengths (red: 633 nm, blue: 473 nm). The left part shows printing images, and the right part is the holographic images. The quality of holographic images in Fig. 4 is better than that shown in this figure.


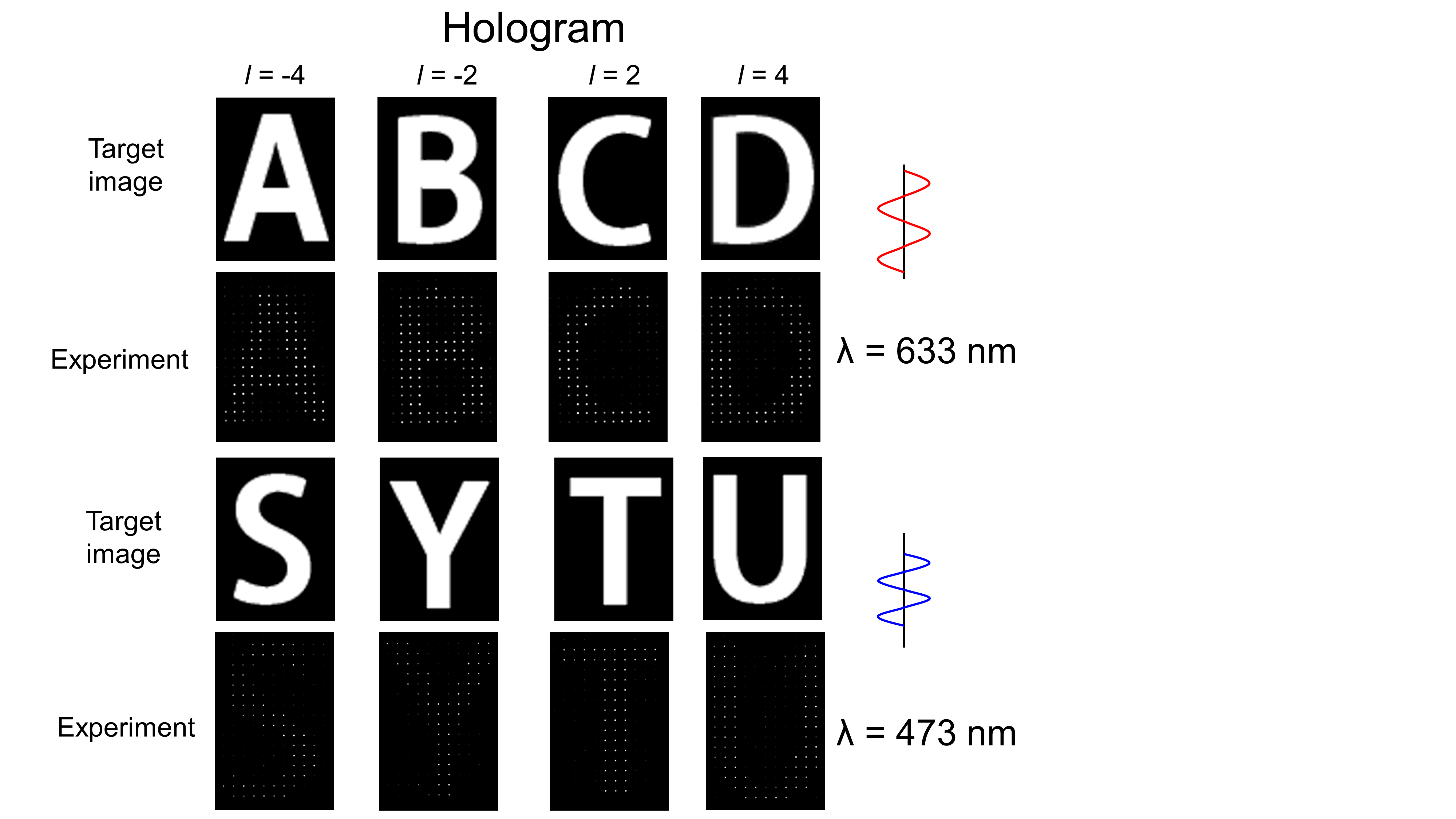


**Supplementary Fig. S6** Demonstration of the OAM holograms. All parameters except pixel count (800*800) and topological charges (*l* = -4, -2, 2, 4) are consistent with the metasurface shown in Fig. 4.


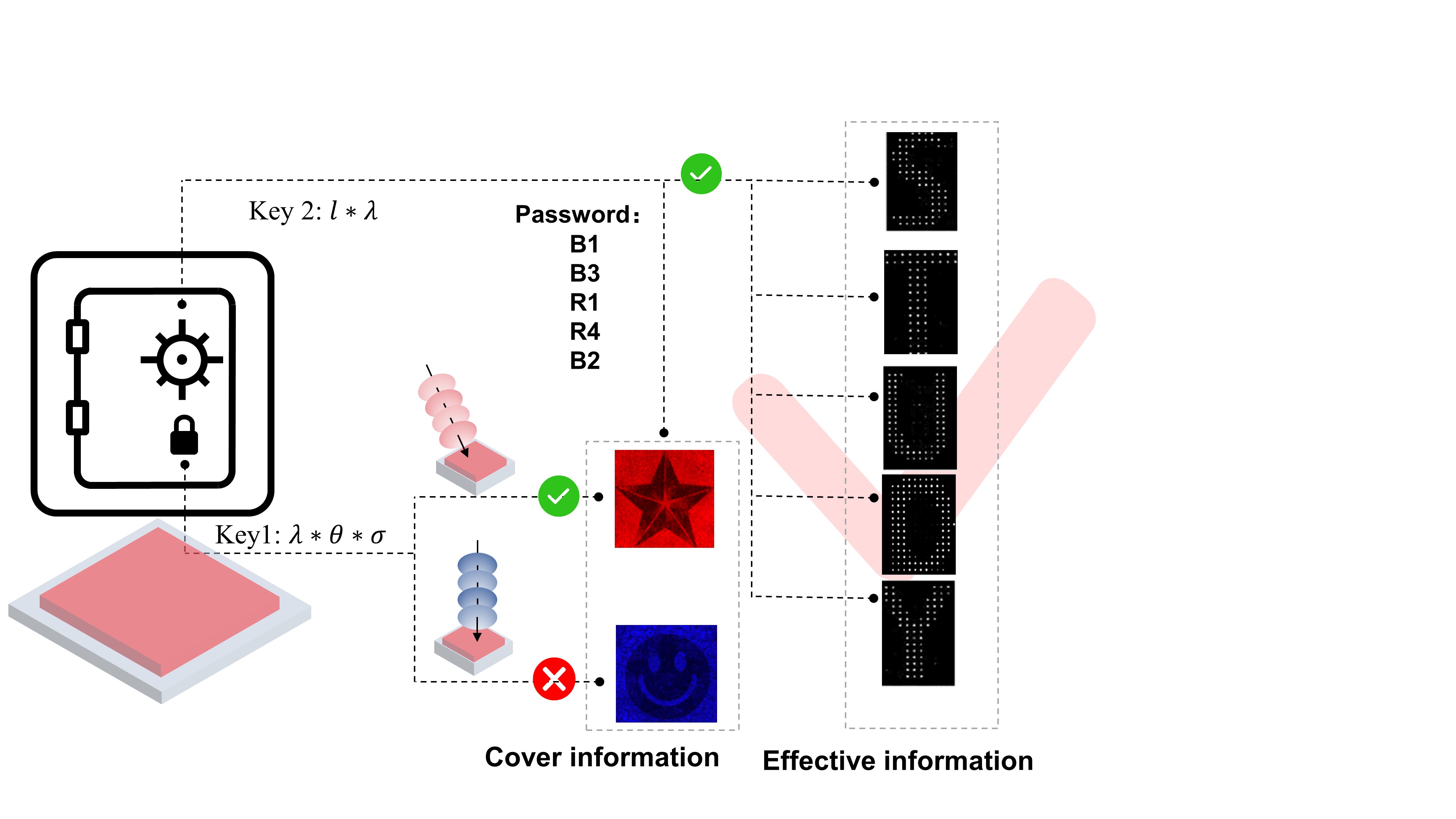


**Supplementary Fig. S7** Diagram of a super-encryption method which is composed of two encryption strategies in one metasurface. To obtain the password for the holographic image, deciphering must be performed using the correct incident angle, wavelength, and polarization, which has high information security. Hence high data capacity and strong encryption can be implemented in one metasurface.


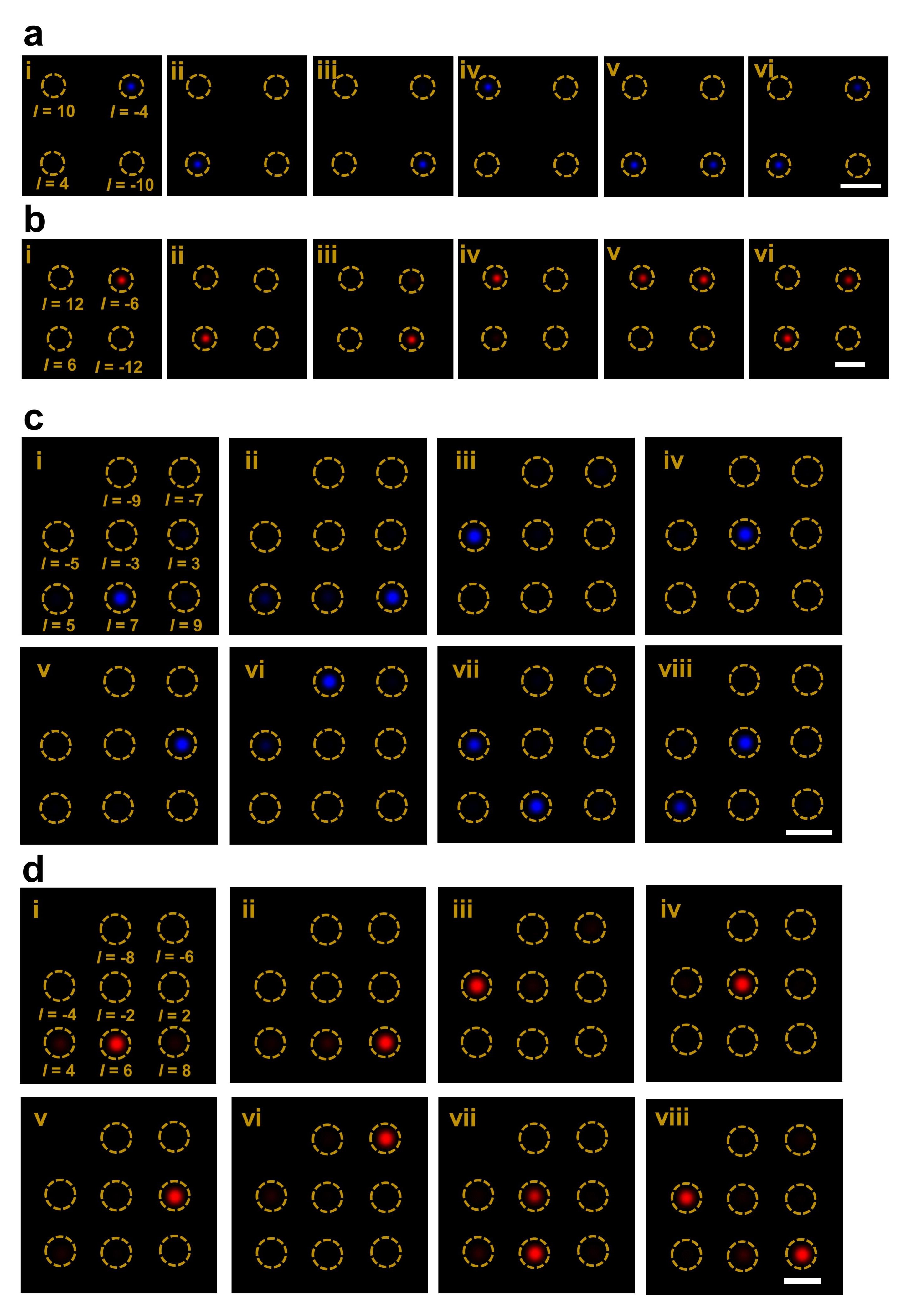


**Supplementary Fig. S8 a** Experimental results of the 8-channels demultiplexer when incident OAM beam is 473 nm. The scale bar is 90 μm. **b** Experimental results of the 8-channels demultiplexer when incident OAM beam is 633 nm. The scale bar is 60 μm. **c** Experimental results of the 16-channels demultiplexor when incident OAM beam is 473 nm. The scale bar is 30 μm. **d** Experimental results of the 16-channels demultiplexor when incident OAM beam is 633 nm. The scale bar is 20 μm. For clear display, the images show the intensity corresponding to the wavelength.


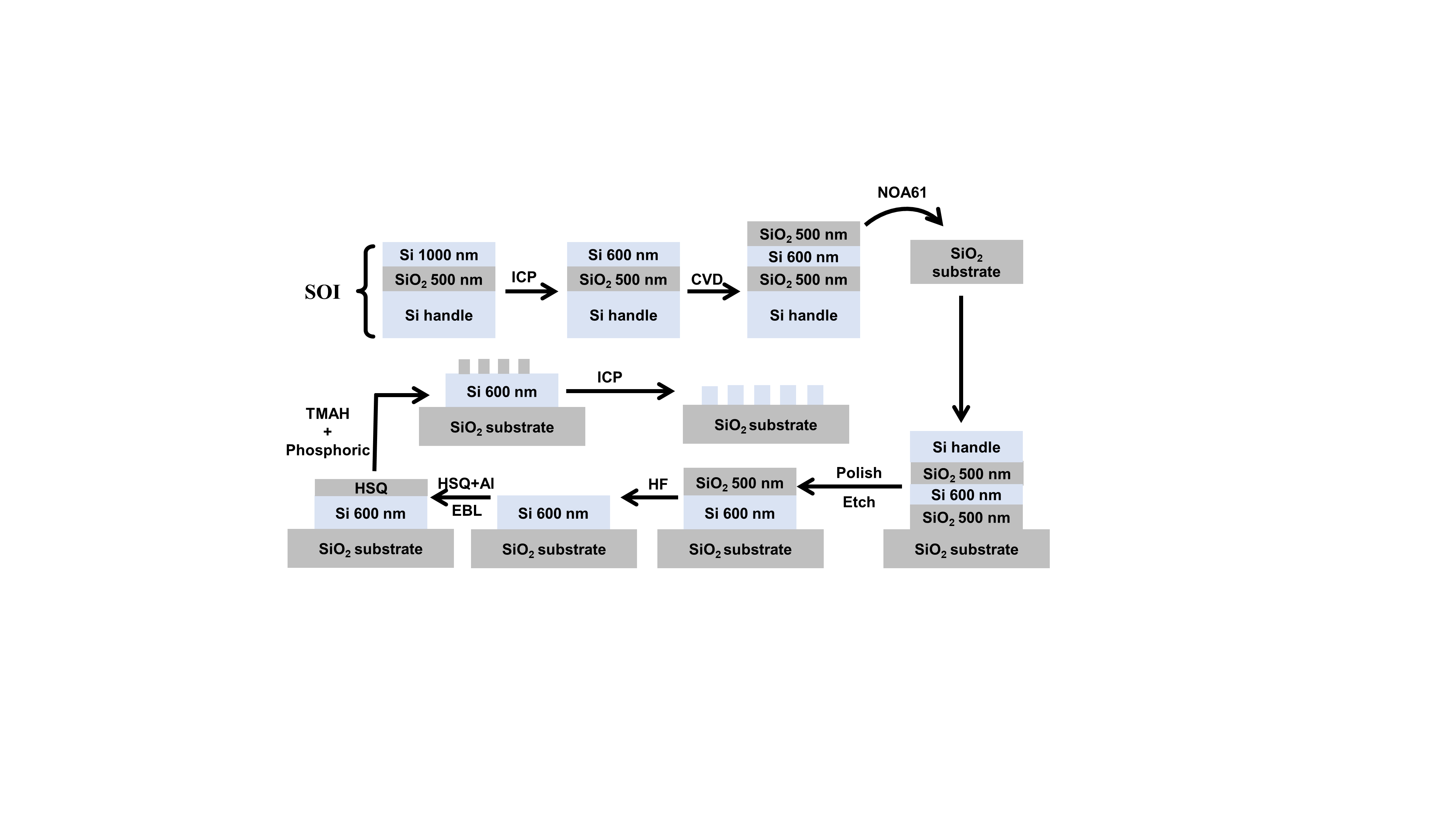


**Supplementary Fig. S9** The flow chart of the entire metasurface fabrication process. A SOI wafer is etched by ICP (HBr), and the thickness of the upper c-silicon layer is reduced from 1000 nm to 600 nm. Next, 500 nm thickness SiO_2_ is deposited on top of the SOI wafer through an inductively coupled plasma chemical vapor deposition (ICP-CVD) process. After adhesive NOA61 is spun onto it, the processed SOI wafer is subsequently inverted onto a SiO_2_ substrate (thickness 1 mm). Then, the excess Si is removed by polish and ICP etching (SF6), and the protective layer (500 nm SiO_2_) is removed using 10% HF acid for about 8 minutes. 140 nm HSQ is spin-coated on the sample with 4000 rpm before baked for 3 minutes at 93 °C. After that, a 30 nm thickness layer of aluminum is deposited using thermal evaporation, which acts as a layer for dissipating charges. Finally, the metasurface pattern is fabricated by electric beam lithography (EBL), and the rest Al and HSQ layer are removed by 5% phosphoric acid and tetramethylammonium hydroxide solutions respectively. The structure is fabricated through ICP (HBr) etching.

**Note S1. Discussion on the situations of 2 nanopillars in a pixel.**

First, we will discuss the response of a single nanopillar in the optical field (Fig. 1a). A relative Cartesian coordinate system is established, and the long axis of the nanopillar is aligned with the x-axis. The center of the nanopillar coincides with the origin. Following the Jones calculus, we can denote the Jones matrix of the nanopillar as:

$$M_{L}\left( 0 \right)=\left[ \begin{matrix} t_{x} & 0 \\ 0 & t_{y} \end{matrix} \right]$$

If the nanopillar rotates around its center with angle *θ*, the above formula can be modified to:

$$M_{L}\left( \theta\right)=\left[ \begin{matrix} \cos\theta& -sin\theta\\ \sin\theta& \cos\theta\end{matrix} \right]M_{L}\left( 0 \right)\left[ \begin{matrix} \cos\theta& \sin\theta\\ -sin\theta& \cos\theta\end{matrix} \right]$$

$$=\left[ \begin{matrix} t_{x}\cos^{2}\theta+t_{y}\sin^{2}\theta& t_{x}\sin\theta\cos\theta-t_{y}\sin\theta\cos\theta\\ t_{x}\sin\theta\cos\theta-t_{y}\sin\theta\cos\theta& t_{x}\sin^{2}\theta+t_{y}\cos^{2}\theta\end{matrix} \right]$$

The eigenstates of the above Jones matrices are linearly polarized states. For clearer understanding, we will transform these matrices using unitary transformations which can change the eigenstates to circularly polarized states:

$M_{\mathrm{cir}}=Ф^{-1}M_{L}Ф$ and Ф=$\frac{1}{\sqrt{2}}\left[ \begin{matrix} 1 & 1 \\ i & -i \end{matrix} \right]$

So $M_{\mathrm{cir}}(\theta)=\frac{1}{2}\left[ \begin{matrix} t_{x}+t_{y} & {(t}_{x}-t_{y})e^{-2i\theta} \\ {(t}_{x}-t_{y})e^{2i\theta} & t_{x}+t_{y} \end{matrix} \right]$

For incident circularly polarized light,$\left[ \begin{matrix} 1 \\ 0 \end{matrix} \right]$, the transmitted optical field can be represented as:

$$M_{\mathrm{cir}}\left( \theta\right)\left[ \begin{matrix} 1 \\ 0 \end{matrix} \right]=\frac{1}{2}\left[ \begin{matrix} t_{x}+t_{y} \\ {(t}_{x}-t_{y})e^{2i\theta} \end{matrix} \right]=\frac{1}{2}\left[ \left( t_{x}+t_{y} \right)\left[ \begin{matrix} 1 \\ 0 \end{matrix} \right]+{(t}_{x}-t_{y})e^{2i\theta}\left[ \begin{matrix} 0 \\ 1 \end{matrix} \right] \right]$$

Hence, the transmitted cross-polarized light can be represented as:

$$E_{\mathrm{out}}=\frac{1}{2}{(t}_{x}-t_{y})e^{2i\theta}\propto e^{2i\theta}$$

Next, we will consider two nanopillars with rotation angles *θ*_1_ and *θ*_2_ as a pixel, namely coherent pixel. Here is the transmission coefficient of the cross-polarized beam through the coherent pixel cell when the incident beam is the other circularly polarized wave:

$$E\propto e^{2i\theta_{1}} +e^{2i\theta_{2}}$$

Using certain trigonometric formulas, the above result can be expressed as:

$$E\propto2\cos\delta e^{i(2\varphi_{1}+\delta)}$$

Therefore, the amplitude and phase of the electric field are manipulated independently by cos*δ* and (2*θ*_1_+*δ*) respectively, where *δ* is the difference of rotation angle between the two nanopillars.

**Note S2. The multiplexing of OAM holographic images.**

The complex-amplitude plane of our metasurface is the sum of inverse Fourier transform results (left part of Fig. 3a) and helical wavefronts, which can be represented as $E_{sum}=\sum_{j=1}^{n} A_{p}e^{i\varphi_{j}}e^{il_{j}\theta}$. In this equation, *i* and *p* represent the imaginary symbol and a certain wavelength channel, and $A_{p}e^{i\varphi_{j}}$ denotes the complex-amplitude distribution corresponding to j^th^ holographic image. $e^{il_{j}\theta}$ represents an additional spiral phase, where *l* and $\theta$ represent the topological charge and azimuthal angle respectively. When optical fields ${(E}_{sum})$ passes through a Fresnel lens, namely Fourier transform, the reconstructed optical fields can be written as $\mathcal{F}\left[ E_{sum} \right]=\sum_{j=1}^{n} \mathcal{F}\left( A_{p}e^{i\varphi_{j}} \right)*\mathcal{F}\left( e^{il_{j}\theta} \right)$, where $\mathcal{F}$ and $*$ are Fourier transform operator and convolution symbol respectively. Next, we discuss the two cases of incident wave: 1) When the incident wave is a plane wave $e^{i\varphi_{0}}$, the above equation doesn't change because $\varphi_{0}$ is a constant. Hence, the reconstructed signal is still a doughnut-shaped intensity distribution. 2) If the incident wave is a vortex wave $e^{il_{k}\theta}$, the equation will be $\mathcal{F}\left[ E_{sum} \right]=\sum_{j=1}^{n} \mathcal{F}\left( A_{p}e^{i\varphi_{j}} \right)*\mathcal{F}\left( e^{i\theta\left( l_{j}+l_{k} \right)} \right)$. Hence, the reconstructed optical fields become a spot only when $l_{j}$ and $l_{k}$ are opposite to each other. If the signal in the middle can pass through, only the reconstructed hologram with a certain topological charge corresponding to the incident wave can be received. The details of the filtering method are shown in Fig. S10. Therefore, the scale bar in holograms can represent the distance between these bright spots rather than the size of the bright spots, which have been convolved with a Gaussian kernel.


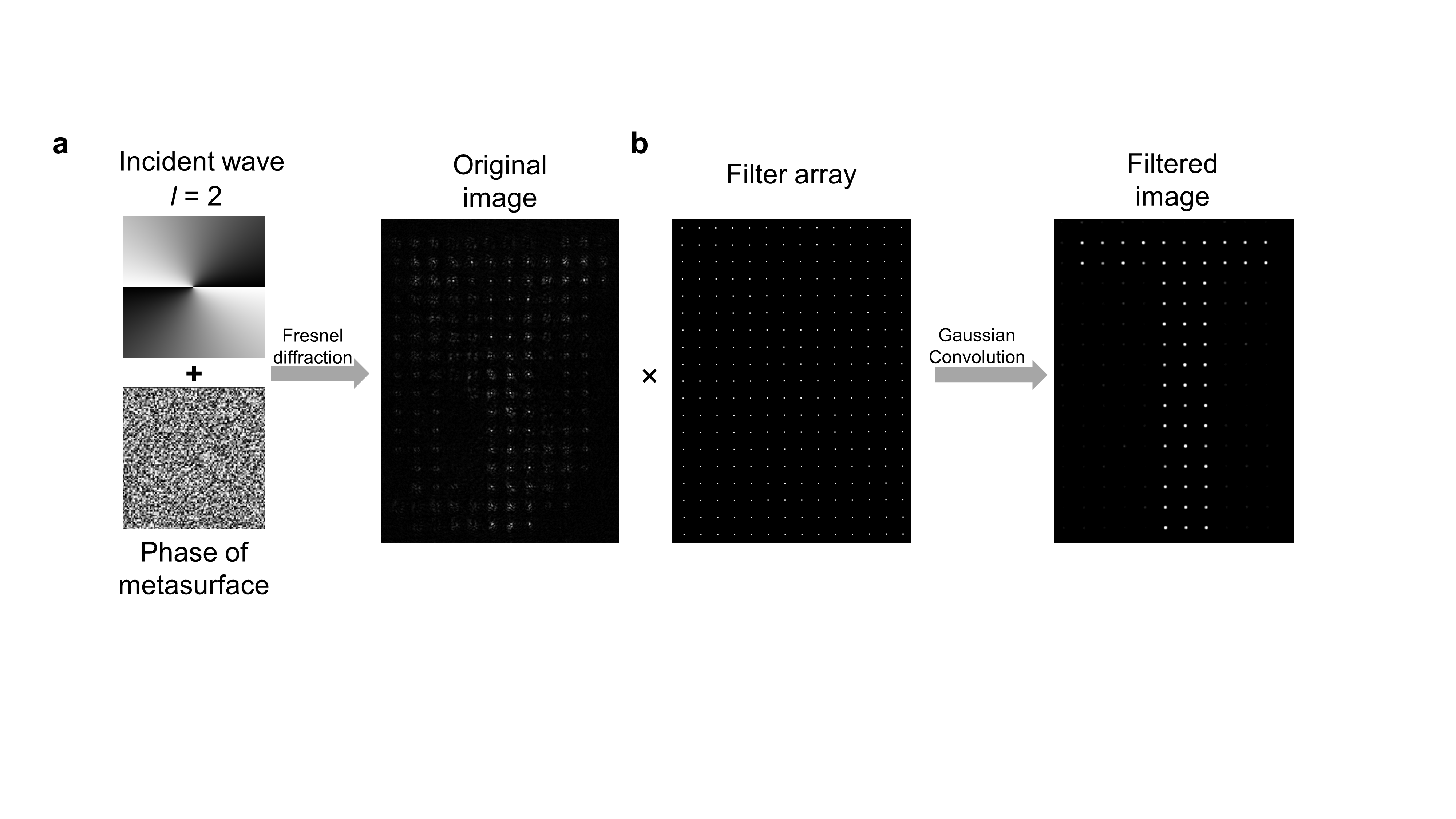


**Supplementary Fig. S10** **a** The process of obtaining the original image. **b** Filtering method for the original signal.

**Note S3. The calculation of sample distance.**

After Fourier transform, the electric field distribution of the OAM beams is determined by the following equation:

$$E\left( \rho\right)=\frac{2\pi{(-1)}^{l+1}}{\lambda f}\int_{0}^{R} J_{l}\left( r\rho\frac{2\pi}{\lambda f} \right)rdr$$

This equation is applicable in cylindrical coordinate. Since the field distribution is radial symmetry, we ignore the azimuth angle in the cylindrical coordinate. R and *l* represent the radius and topological charge of the OAM beams respectively. λ and *f* are the wavelength of the incident wave and the focal length of the Fresnel lens. r and ρ stand for the radius in the incident plane and focal plane. J is the Bessel function.

If we define the region where the intensity is greater than 0.1 as non-overlapping area, Fig S11 shows the interval of sample array as a function of topological charge |*l*| with two wavelengths (R = 480 μm, red: *λ* = 633 nm, *f* = 1.6 mm; blue: *λ* = 473 nm, *f* = 2.9 mm).


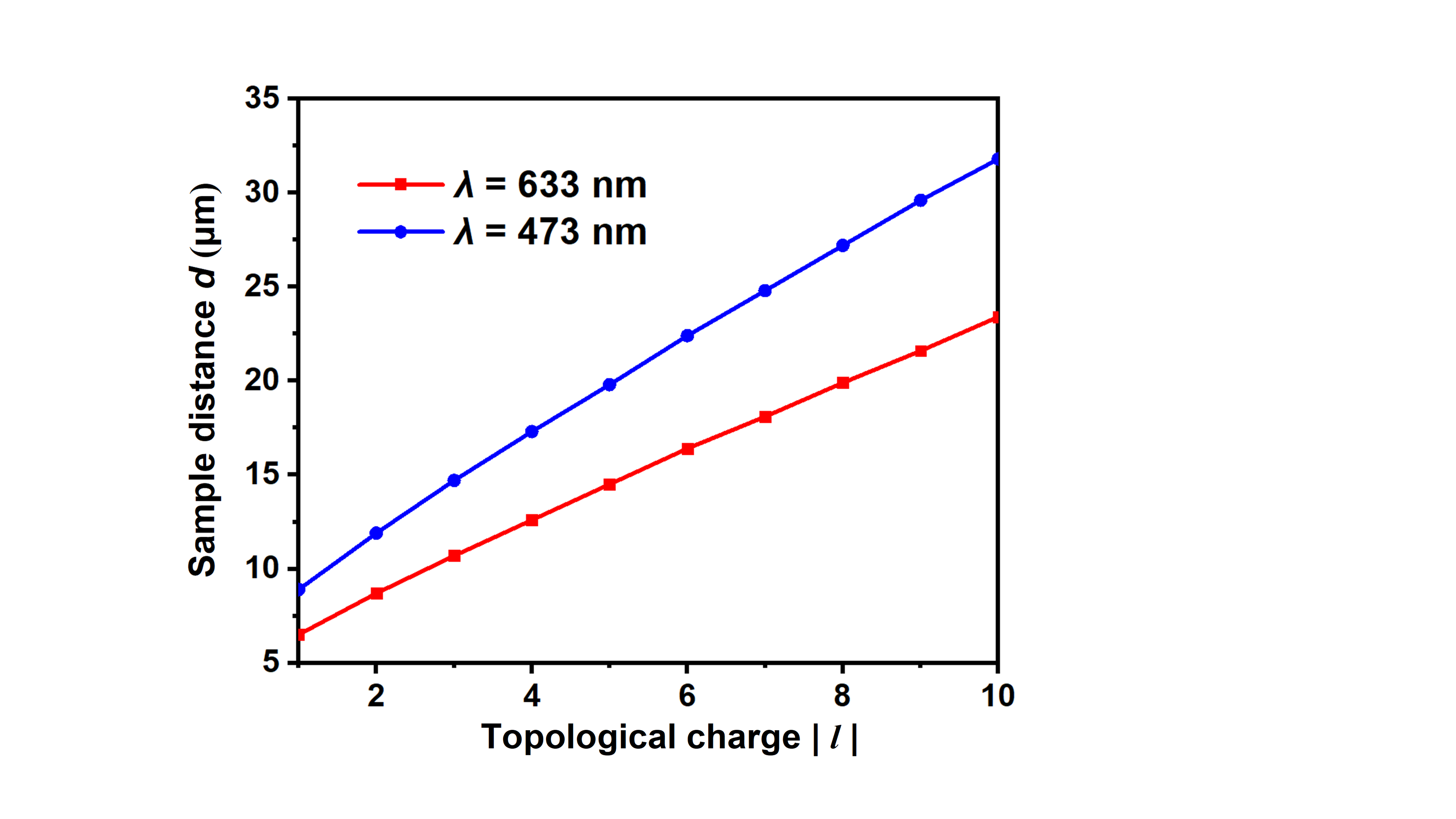


**Supplementary Fig. S11** Numerical characterization of sample distance (d) as a function of topological charge |*l*| with different wavelengths (red: 633 nm, blue: 473 nm). The lenses are both 480 μm in diameter but have different focal distances (red: 1.6 mm, blue: 2.9 mm). The interval of sample array, namely sample distance, is the critical design parameter which affect the limit of the number of different holographic images. If we need to increase the highest order of topological charges in a metasurface, i.e., the upper limit in the number of different images, we must increase the interval of sample array to avoid the overlapping between different signals in momentum space. In addition, the interval of topological charge and the size of the metasurface also influence the quantity of the integrated images, since these two parameters affect the radius of the OAM signals in momentum space.
